# Pharmacological Treatment of Idiopathic Pulmonary Fibrosis: Current Approaches, Unsolved Issues, and Future Perspectives

**DOI:** 10.1155/2015/329481

**Published:** 2015-12-08

**Authors:** Michael Kreuter, Francesco Bonella, Marlies Wijsenbeek, Toby M. Maher, Paolo Spagnolo

**Affiliations:** ^1^Center for Interstitial and Rare Lung Diseases, Pneumology and Respiratory Critical Care Medicine, Thoraxklinik, University of Heidelberg, 69126 Heidelberg, Germany; ^2^Translational Lung Research Center Heidelberg (TLRCH), Member of the German Center for Lung Research (DZL), 69126 Heidelberg, Germany; ^3^Interstitial and Rare Lung Disease Unit, Ruhrlandklinik, University Hospital, University of Duisburg-Essen, 45141 Essen, Germany; ^4^Department of Pulmonary Disease, Erasmus Medical Centre, University Hospital Rotterdam, 3015 CE Rotterdam, Netherlands; ^5^National Institute for Health Research Biological Research Unit, Royal Brompton Hospital, London SW3 6NP, UK; ^6^National Heart and Lung Institute, Imperial College, London SW3 6NP, UK; ^7^Medical University Clinic, Canton Hospital Baselland and University of Basel, 4410 Liestal, Switzerland

## Abstract

Idiopathic pulmonary fibrosis (IPF) is a devastating condition with a 5-year survival of approximately 20%. The disease primarily occurs in elderly patients. IPF is a highly heterogeneous disorder with a clinical course that varies from prolonged periods of stability to episodes of rapid deterioration. In the last decade, improved understanding of disease mechanisms along with a more precise disease definition has allowed the design and completion of a number of high-quality clinical trials. Yet, until recently, IPF was essentially an untreatable disease. Finally, pirfenidone and nintedanib, two compounds with antifibrotic properties, have consistently proven effective in reducing functional decline and disease progression in IPF. This is a major breakthrough for patients and physicians alike, but there is still a long way to go. In fact, neither pirfenidone nor nintedanib is a cure for IPF, and most patients continue to progress despite treatment. As such, comprehensive care of patients with IPF, including management of comorbidities/complications and physical debility and timely referral for palliative care or, in a small number of highly selected patients, lung transplantation, remains essential. Several agents with high potential are currently being tested and many more are ready to be evaluated in clinical trials.

## 1. Disease Overview

Idiopathic pulmonary fibrosis (IPF), the most common of the idiopathic interstitial pneumonias (IIPs) and one of the most prevalent interstitial lung diseases (ILDs), is a chronic, progressive, and almost invariably fatal disorder characterised by scarring of the lung and progressive loss of function [1, 2]. The disease, which is limited to the lungs and occurs primarily in older adults, is associated with a radiological and/or histopathologic pattern of usual interstitial pneumonia (UIP) [1], which, however, is not unique to IPF, but can be found in a number of conditions, including connective tissue diseases (CTDs), chronic hypersensitivity pneumonitis (HP), and asbestosis. Accordingly, the diagnosis of IPF requires the exclusion of all known causes of fibrotic interstitial pneumonia and the presence of an* idiopathic* UIP pattern [1]. IPF is an age-related condition; indeed, the disease is unlikely to occur in individuals younger than 50 but is present in approximately 0.2% of those older than 75 [3, 4]. The incidence of IPF, estimated at 3–9 cases per 100,000 per year in Europe and North America, is increasing worldwide [5], and IPF is also likely to account for much of the increased ILD-related mortality reported worldwide between 1990 and 2013 [6]. Although the nature of the stimuli that trigger the fibrotic process in IPF is still unknown, several factors have been associated with an increased risk of developing the disease, including cigarette smoking, environmental/occupational pollutants, microbial agents, chronic microaspiration secondary to gastroesophageal reflux, and genetic abnormalities [7–9]. Despite its inexorably progressive nature with a 5-year survival of approximately 20% in historical cohorts [10–13], IPF is characterized by a highly variable clinical course (e.g., periods of relative stability may be punctuated by episodes of acute worsening often resulting in respiratory failure and death), which makes the natural history of the disease largely unpredictable in individual patients [14].

The understanding of IPF pathobiology has substantially improved over time, and this has shifted the approach to treatment. Based on the traditional (and erroneous) hypothesis that inflammation preceded fibrosis in IPF, the 2000 statement recommended corticosteroids in addition to cytotoxic agents (either azathioprine or cyclophosphamide) as the “standard treatment,” although limited and low-quality data supported this recommendation [15]. Conversely, current concepts suggest that on-going microinjury to alveolar epithelial cells induces a fibrotic environment and that growth factors secreted by the injured epithelial cells promote fibroblast recruitment, proliferation, and differentiation to invasive myofibroblasts [16]. In turn, actively proliferating fibroblasts and myofibroblasts organize into* fibroblastic foci* and are responsible for the excessive collagen production that results in scarring of the lung and architectural distortion [16, 17]. More recently, clinical trials have therefore shifted their focus from anti-inflammatory and immunosuppressant drugs to newer molecules with antifibrotic properties. Nevertheless, most of these studies have been negative, probably due to the multitude of mediators, growth factors, and signalling pathways involved in the fibrotic process of IPF. Accordingly, no pharmacological treatment was recommended by the 2011 guidelines; the only care options recommended were palliation and, in highly selected patients, lung transplantation [1].

Since the publication of the 2011 guideline, two antifibrotic compounds, namely, pirfenidone and nintedanib, have proven effective in reducing functional decline and disease progression in IPF [18–20], whereas warfarin and the three-drug regimen prednisone/azathioprine/N-acetylcysteine, which in the 2011 document had been given a* weak* recommendation* against* use, have subsequently been shown to be harmful in patients with IPF [21, 22]. An update of the 2011 Clinical Practice Guideline has therefore been recently published [23]. In this paper, we summarize the most recent advances in pharmacological treatment of IPF and discuss challenges and future perspectives in the management of this devastating disease.

## 2. The Updated 2011 Clinical Practice Guideline

The current guideline on management of IPF is evidence-based and includes quality and grading of the evidence. For any given question, the panel of experts graded the quality of the evidence available (e.g., high, moderate, low, or very low, with the study designs of the highest quality of evidence being randomized control trials (RCTs) and lowest case reports and case series) and made a recommendation* for* or* against* a given intervention. Recommendations were decided on the basis of majority vote and were either “strong” or “weak/conditional” [1, 23]. However, while in the 2011 guideline both conflicted and nonconflicted members were allowed to participate without restrictions to all the key steps of the recommendation formulating process, in the 2015 update only committee members* without* conflicts of interest had the opportunity to vote on the recommendations [23]. Notably, the exclusion of conflicted members from voting on the recommendations, which was dictated by the need to minimize potential bias, has probably resulted in the paradox of pirfenidone, nintedanib, and antiacid therapy which have being given the same recommendation (e.g., conditional/weak recommendation* for* use) despite different levels of evidence (discussed later) [23]. No treatment received a “strong yes” recommendation. In other words, no treatment is strongly recommended by current guideline for patients with IPF. On the other hand, several pharmacological treatments received a strong recommendation* against* use, mostly due clear evidence of inefficacy.  Table 1 summarizes the key recommendations on pharmacological treatment of IPF according to current guideline.

In the following section, only interventions that received a* conditional* recommendation* for* use or a* conditional* recommendation* against* use are reviewed (Table 2).

### 2.1. Pirfenidone

Pirfenidone is an orally available, synthetic pyridone compound. Although its mechanism of action is not fully understood,* in vitro* studies and animal models of pulmonary fibrosis suggest that pirfenidone exerts antifibrotic, anti-inflammatory, and antioxidant properties through downregulation of key profibrotic growth factors, including transforming growth factor- (TGF-) *β*; inhibition of cytokines and inflammatory cells; and scavenge of reactive oxygen species (ROS), respectively [24, 25]. The efficacy of pirfenidone in patients with IPF has been evaluated in four randomized, double-blind, placebo-controlled, phase 3 clinical trials, one conducted in Japan [26] and three multinational [18, 27]. The CAPACITY (Clinical Studies Assessing Pirfenidone in Idiopathic Pulmonary Fibrosis: Research of Efficacy and Safety Outcomes) program consisted of two almost identical studies (PIPF-004 and PIPF-006) and enrolled patients aged 40–80 years with mild-to-moderate functional impairment (forced vital capacity (FVC) > 50% predicted and diffusion capacity of the lung for carbon monoxide (DL_CO_) > 35% predicted). In study 004, 435 patients were assigned in a 2 : 1 : 2 dosing ratio to pirfenidone 2403 mg/day, pirfenidone 1197 mg/day, or placebo, whereas study 006 enrolled 344 patients who were assigned in a 1 : 1 ratio to pirfenidone 2403 mg/day or placebo. The primary endpoint of change in percentage predicted FVC from baseline to week 72 was met in study 004 (e.g., mean FVC change at week 72 was −8.0% in the pirfenidone 2403 mg/day group and −12.4% in the placebo group; *p* = 0.001), whereas no significant difference was observed between pirfenidone and placebo in study 006. The CAPACITY trials provided mixed results also for the secondary endpoints. Indeed, pirfenidone significantly reduced the proportion of patients with an FVC decline ≥ 10% and significantly prolonged progression-free survival (PFS) (defined as time to confirmed ≥ 10% decline in percentage predicted FVC, ≥15% decline in percentage predicted DL_CO_ or death) compared with placebo in study 004, but not in study 006. Conversely, pirfenidone significantly reduced the decline in 6-minute walk test (6MWT) distance compared with placebo in study 006, but not in study 004. A Cochrane systematic review and meta-analysis showed that pirfenidone significantly reduced the rate of functional decline and disease progression (defined as time to confirmed ≥10% decline in percentage predicted FVC/vital capacity (VC) or death) compared with placebo [28].

In 2011 pirfenidone, which had already been approved in Japan in 2008, was approved in Europe, whereas the US Food and Drug Administration (FDA) denied approval and requested an additional phase 3 study to provide supportive evidence of efficacy. In ASCEND (Assessment of Pirfenidone to Confirm Efficacy and Safety in Idiopathic Pulmonary Fibrosis), 555 IPF patients were randomized 1 : 1 to pirfenidone 2403 mg/day (*n* = 278) or placebo (*n* = 277) [18]. The study met its primary outcome of change from baseline to week 52 in the percentage of predicted FVC (−164 mL in the pirfenidone arm versus −280 mL in the placebo arm; absolute difference 116 mg, relative difference 41.5%; *p* < 0.0001). In addition, pirfenidone reduced by 47.9% the proportion of patients with a decline of ≥10% in percentage predicted FVC or who died and increased the proportion of patients who had no decline in percentage predicted FVC compared with placebo (16.5% versus 31.8%, *p* < 0.001, and 22.7% versus 9.7%, *p* < 0.001, resp.). Additional beneficial effects of pirfenidone included a reduction in the decline of the 6MWT distance (*p* = 0.036) and improved PFS (defined as the time to the first occurrence of any one of the following: a confirmed decrease of 10% or more in the predicted FVC, a confirmed decrease of 50 meters or more in the 6MWT distance, or death). Moreover, a prespecified pooled analysis including also data from the two CAPACITY studies showed that pirfenidone compared with placebo significantly reduced both all-cause mortality (3.5% versus 6.7%, resp.; *p* = 0.01, HR: 0.52) and IPF-related mortality (1.1% versus 3.5%, resp.; *p* = 0.006, HR: 0.32) at 52 weeks. Commonly reported treatment-related adverse events included nausea, dyspepsia, dizziness, vomiting, rash, photosensitivity reaction, anorexia, insomnia, and abdominal distension, which however were mild-to-moderate in severity and reversible and without clinically significant sequelae. Recommendations on optimal management of pirfenidone-related adverse events based on existing guidelines and expert opinion have recently been published [29]. The US FDA approved pirfenidone for the treatment of IPF in October 2014. The 2015 guideline gives a conditional recommendation for use of pirfenidone in patients with IPF (e.g., “this recommendation puts a high value on the potential benefit of pirfenidone on patient-important outcomes such as disease progression as measured by rate of FVC decline and mortality and a lower value on potentially significant adverse effects and the cost of treatment”) [23].

### 2.2. Nintedanib

Nintedanib, an orally administered inhibitor of multiple tyrosine kinases originally developed as an anticancer drug, is a small molecule that targets vascular endothelial growth factor receptors (VEGFR) 1–3, fibroblast growth factor receptors (FGFR) 1–3, and platelet-derived growth factor receptors (PDGFR) *α* and *β* [30]. VEGF, FGF, and PDGF mediate a number of processes including fibrogenesis and angiogenesis and have been implicated in the pathogenesis of IPF [31]. The efficacy of nintedanib in patients with IPF has been evaluated in two identical, randomized, double-blind, placebo-controlled, multinational, 52-week duration, phase 3 trials (INPULSIS-1 and INPULSIS-2) [19]. A total of 1,066 patients were randomly assigned in a 3 : 2 ratio to receive nintedanib 150 mg twice daily (*n* = 309 in INPULSIS-1 and *n* = 329 in INPULSIS-2) or placebo (*n* = 204 in INPULSIS-1 and *n* = 219 in INPULSIS-2). Inclusion criteria included age ≥ 40 years, diagnosis of IPF made within the previous 5 years, FVC of ≥50% of predicted value, DL_CO_ of 30–79% of predicted value, and chest high-resolution computed tomography (HRCT) performed within the previous 12 months. Notably, in the absence of a confirmatory surgical lung biopsy, the INPULSIS trials allowed the enrolment of patients with a radiological pattern of* possible* UIP [32]. In both trials nintedanib significantly reduced the rate of decline in FVC over the study period (the primary end point). In fact, the annual rate of decline in FVC was −114.7 mL in the nintedanib arm and −239.9 mL in the placebo arm in INPULSIS-1 (difference: 125.3 mL; *p* < 0.001) and −113.6 mL and −207.3 mL in INPULSIS-2 (difference: 93.7 mL; *p* < 0.001), respectively. Prespecified sensitivity analyses, in particular the multiple imputation analyses of missing data, confirmed that the results of the primary analysis were robust [19]. In addition, in both trials patients in the nintedanib arm were significantly more likely than patients in the placebo arm to be functionally stable at week 52 (e.g., to have no absolute decline in percent predicted FVC > 5%; 52.8% versus 38.2%, *p* = 0.001 in INPULSIS-1, and 53.2% versus 39.3%, *p* = 0.001 in INPULSIS-2, resp.). As for the key secondary end points, the two trials provided mixed results in terms of both St. George's Respiratory Questionnaire (SGRQ), which was significantly improved in INPULSIS-2 (*p* = 0.02) but not in INPULSIS-1 (*p* = 0.97), and time to first acute exacerbation (AE), which was statistically significant in INPULSIS-2 (*p* = 0.005) but not in INPULSIS-1 (*p* = 0.67). However, based on the results of a prespecified sensitivity analysis of pooled data from INPULSIS-1 and INPULSIS-2, the time to first adjudicated AE (either “confirmed” or “suspected” by an expert adjudication panel) was significantly increased in the nintedanib arm compared to placebo (*p* = 0.001). Conversely, in a prespecified pooled analysis, the adjusted mean change from baseline in the SGRQ total score did not differ significantly between the nintedanib and placebo groups [19]. In addition, in a prespecified pooled analysis, a nonstatistically significant trend favouring nintedanib was observed in all-cause mortality (5.5% in the nintedanib arm versus 7.8% in the placebo arm, resp.; *p* = 0.14, HR: 0.70) or death from a respiratory cause (3.8% in the nintedanib arm versus 5.0% in the placebo arm, resp.; *p* = 0.34, HR 0.74). Overall, nintedanib showed an acceptable safety and tolerability profile as shown by the comparable incidences of adverse events across all groups [19]. The most common drug-related side effects were diarrhea, nausea, and vomiting. Among nintedanib and placebo recipients, diarrhea was reported by 61.5% and 18.6% of patients in INPULSIS-1 and 63.2% and 18.3% of patients in INPULSIS-2, respectively. However, over 90% of cases of diarrhea were mild-to-moderate. Nintedanib has recently been approved both in USA and Europe for the treatment of IPF.

The 2015 guideline gives a conditional recommendation for use of nintedanib in patients with IPF (e.g., “this recommendation puts a high value on the potential benefit of nintedanib on patient-important outcomes such as disease progression as measured by rate of FVC decline and mortality and a lower value on potentially significant adverse effects and the expected cost of treatment”) [23].

### 2.3. Antiacid Therapy

Abnormal gastroesophageal reflux (GER), including clinically silent GER, is highly prevalent in patients with IPF and chronic microaspiration secondary to GER is considered a risk factor for the development or worsening of the disease [33]. In an uncontrolled retrospective study of 204 IPF patients, antiacid treatment was associated with reduced radiological fibrosis and longer survival time [34]. In a more recent analysis of patients assigned to placebo arms in three IPFnet-sponsored RCTs of different pharmacological interventions (*n* = 242), patients taking antiacid treatment at baseline (either proton-pump inhibitors or H_2_ blockers) had a smaller decrease in FVC at 30 weeks compared with those not taking antiacid treatment (*p* = 0.05) after adjusting for sex, baseline percentage predicted FVC, and baseline percentage predicted DL_CO_ [35]. This study showed no benefit of antiacid therapy on all-cause mortality or all-cause hospitalization. Despite the lack of data from prospective RCTs, the 2015 guidelines give a conditional recommendation for use of antiacid therapy in patients with IPF (e.g., this recommendation places a higher value on possible improved lung function and survival and the low cost of therapy and a lower value on the potential increased risk for pneumonia) [23]. However, the panel acknowledged the need for further research focusing on efficacy (e.g., RCTs of antiacid therapy versus placebo) and long-term safety of antiacid therapy as well as interactions with other IPF medications.

### 2.4. Sildenafil

Sildenafil, an oral phosphodiesterase-5 inhibitor that stabilizes the second messenger of nitric oxide (e.g., cyclic guanosine monophosphate) leading to pulmonary vasodilatation, is approved for treatment of pulmonary arterial hypertension (PAH) both as monotherapy and in combination with other drugs [36]. Following promising results from a small open-label study [37], the STEP-IPF (Sildenafil Trial of Exercise Performance in Idiopathic Pulmonary Fibrosis) trial enrolled 180 patients with advanced IPF (DL_CO_ < 35% predicted) who were randomized to either sildenafil (20 mg three times daily) or placebo for 12 weeks, with a subsequent 12-week open-label phase during which all patients received the active drug [38]. The study did not meet its primary outcome, which was the proportion of patients whose 6MWD improved by ≥20 meters. However, significant differences favoring sildenafil were observed in some secondary outcomes (e.g., arterial oxygen saturation, DL_CO_, degree of dyspnea, and quality of life). In addition, a predefined subgroup analysis of 119 patients with available echocardiogram data showed that in patients with echocardiographic evidence of right ventricular systolic dysfunction sildenafil treatment was associated with a significant improvement in the primary outcome of 6MWD [39]. The recommendation on sildenafil use in patients with IPF given by the recent guideline (e.g., conditional recommendation against use) does not apply to patients receiving phosphodiesterase inhibitors for approved indications such as PAH or other forms of right ventricular dysfunction [23]. The guideline makes no specific recommendation for the subset of patients with IPF and documented PH. Conversely, the recent guidelines on PAH do not recommend the use of sildenafil for treatment of IPF-associated pulmonary hypertension (PH) and suggest to refer patients with IPF-associated PH to highly specialized centers [23, 36].

### 2.5. Dual Endothelin Receptor Antagonists

The effect of bosentan and macitentan, two dual endothelin receptor antagonists (ERAs), in patients with IPF has been examined in two RCTs and one RCT, respectively. In the BUILD-1 (Bosentan Use in Interstitial Lung Disease) study, 158 patients were randomly assigned to receive either oral bosentan (*n* = 74) or placebo (*n* = 84) and followed up for 12 months [40]. While the study failed to meet its primary efficacy endpoint (change in 6MWD by month 12), a treatment effect favoring bosentan (e.g., delayed time to death or disease progression and improvement in quality of life) was observed in a subset of patients diagnosed by surgical lung biopsy. However, the follow-up study (BUILD-3), which specifically enrolled patients diagnosed histologically (*n* = 616), did not show an effect of bosentan on time to disease worsening (defined by a decline ≥10% in FVC and ≥15% in DL_CO_ or an acute exacerbation of IPF at month 12) or death, the primary endpoint [41].

The efficacy and safety of macitentan in IPF patients have been evaluated in a prospective, randomized, double-blind, multicentre, parallel-group, placebo-controlled, phase 2 proof-of-concept study (Macitentan Use in an Idiopathic Pulmonary Fibrosis Clinical (MUSIC) trial) [42]. Of the 178 randomized patients, 119 were allocated to macitentan and 59 to placebo. The study did not meet its primary endpoint (change from baseline up to month 12 in FVC). Similarly, no differences were observed between treatment groups in any of the secondary or exploratory measures, including time to IPF worsening or death. Given the relatively similar mechanism of action between these two compounds and the consistent results, these three studies were pooled for analysis [23]. While no effect between groups was observed in FVC change or mortality, the composite outcome of death or disease progression appeared improved (relative risk: 0.85). The recommendation on bosentan and macitentan use in IPF made by the recent guideline (e.g., conditional recommendation against use) applies to the whole population of patients and there is no specific recommendation for patients with IPF and documented PH. Conversely, the recent guidelines on PAH do not recommend the use of ERAs for treatment of IPF-associated PH and strongly encourage to refer these patients to highly specialized centers for IPF and PH [23, 36].

### 2.6. N-Acetylcysteine

N-acetylcysteine (NAC), a precursor of the endogenous antioxidant glutathione (GSH), has been used in IPF based on the assumption that an oxidant-antioxidant imbalance plays a role in the pathogenesis of the disease [43]. To date, three RCTs of NAC monotherapy in IPF have been performed. In a small pilot study, Tomioka and colleagues randomized 30 patients with IPF to either aerosolized NAC or bromhexine hydrochloride for 12 months [44]. They observed that NAC treatment significantly reduced extent of ground glass on HRCT and KL-6 levels. In a subsequent multicenter, prospective study, 76 Japanese patients were randomly assigned to receive either inhaled NAC or no treatment for 48 weeks [45]. The study did not meet the primary endpoint of change from baseline in FVC during the study period, although NAC treatment was associated with stability in FVC in a subset of patients with early disease (e.g., FVC < 95% of predicted and DL_CO_ < 55% of predicted). The third study was actually part of the IPFnet-sponsored PANTHER (Prednisone, Azathioprine, and N-acetylcysteine: A Study That Evaluates Response in IPF) trial, which had been originally designed to compare three therapeutic interventions, combination prednisone, azathioprine, and high-dose NAC (600 mg three times daily), NAC alone, or placebo [21]. However, following a prespecified efficacy and safety interim analysis planned at approximately 50% of data collection, the combination therapy arm was terminated due to major safety concern, and the study continued as a two-arm study only (NAC monotherapy versus placebo). Compared with placebo, NAC offered no significant benefit in terms of preservation of FVC in patients with IPF [46]. Similarly, no significant between-group differences were observed in the rates of death or acute exacerbations. A pooled analysis of these three studies did not reveal significant benefit of NAC monotherapy on mortality, change in FVC, or quality of life, and both interventions (inhaled and oral NAC) have been given a conditional recommendation against use [23]. However, it has been recently shown that approximately 25% of patients with IPF (those carrying the* TOLLIP* rs3750920 TT genotype) may benefit from NAC therapy, whereas those with the rs3750920 CC genotype may be more susceptible to treatment-related harm [47]. Future studies are needed to confirm whether polymorphisms within* TOLLIP* gene modify the effect of antioxidant therapy in patients with IPF.

## 3. Open Questions on the Pharmacologic Treatment of IPF

### 3.1. Should We Start Antifibrotic Treatment as Early as Possible?

The US FDA has approved both pirfenidone and nintedanib with no prescription limitations and regardless of severity of lung function impairment, although clinical trials have limited enrollment to patients with mild-to-moderate IPF. Once the diagnosis of IPF has been established, it conceptually makes sense to start treatment as early as possible in order to preserve pulmonary function and prolong survival [48].

In a small minority of patients who are asymptomatic and have marginal or no lung function impairment, however, it may not be unreasonable to refrain from starting treatment and adopt a close clinical/functional surveillance after careful evaluation of the risks and benefits of such approach and considering the unpredictable course of IPF.

### 3.2. Which Drug Should We Use as First-Line Treatment for IPF?

Both pirfenidone and nintedanib have been demonstrated to slow the decline in FVC in IPF patients with mild-to-moderate functional impairment with acceptable safety profiles. However, the data available from clinical trials along with the lack of head-to-head comparison between the two drugs do not allow establishing whether one antifibrotic agent is superior over the other and should therefore be used first. The major problem of any head-to-head comparison between pirfenidone and nintedanib is related to the characteristics of the cohorts investigated in the trials. In fact, the inclusion and exclusion criteria of ASCEND, CAPACITY, and INPULSIS trials were different in several ways and resulted in slightly different study populations being enrolled [18, 19, 27]. It is therefore difficult, if at all possible, to compare the different trials to determine better efficacy of these drugs. Moreover, the new guideline did not consider additional evidence of efficacy (e.g., improved survival and reduced risk of acute exacerbations with pirfenidone and nintedanib, resp.) when formulating their recommendations [23]. This was mainly due to methodological issues such as heterogeneity in reporting the study results and statistical power of the single study. The decision as to which agent to use as first-line treatment should therefore be based not only on drug efficacy but also on safety profile as well as patient preference, medical history, concomitant medication, and lifestyle.

### 3.3. How Do We Treat More Severe IPF?

The complexity of the diagnostic process in IPF makes misdiagnosis and delayed diagnosis common [49]. Therefore, it is not infrequent in clinical practice that IPF patients present with advanced disease and severe functional impairment (percentage of the predicted FVC < 50%). While no definitive staging system exists for assessing the severity of the disease, most clinical trials performed to date, including those of pirfenidone and nintedanib, have limited their enrolment to patients with mild-to-moderate functional impairment (FVC ≥ 50% predicted, DL_CO_ ≥ 30–35%). The assumptions behind such restricted inclusion criteria are that patients with advanced disease are less likely to respond to treatment (also because of the frequent coexistence of severe PH in this setting) and may have different and unpredictable response rates and more frequent and severe adverse events, although there are no convincing data in this regard [50]. Therefore, although the FDA has approved both pirfenidone and nintedanib regardless of disease severity (and nintedanib has also been approved by the European Medicines Agency with the same indication), we do not know whether pirfenidone and nintedanib are safe and efficacious also in patients with a more severe functional impairment (e.g., FVC < 50%) as they have never been tested in this patient subset. For patients with DL_CO_ < 35% predicted, echocardiographic evidence of right ventricular dysfunction, and no contraindications to the drug, a trial of sildenafil may be a reasonable therapeutic option.

### 3.4. How Do We Treat Patients with IPF and Lung Cancer?

Patients with IPF are at increased risk of developing lung cancer. The exact prevalence of this complication is unknown but is likely to range between 5% and 17% [51, 52]. Risk factors for lung cancer in IPF include increasing age, male gender, and greater smoking history [53]. Squamous cell carcinoma is the predominant cell type of cancer and tends to localize at the lung periphery and bases (in striking contrast to what is seen in non-IPF patients) and in close proximity to the fibrosis, which may potentially be relevant to the pathogenesis of the neoplastic lesion [54]. While the majority of IPF patients die from progressive disease and respiratory failure, lung cancer has been reported to account for as many as 10% of deaths [55]. In addition, recent data show that the development of this complication has significant prognostic implications [56, 57]. Whether (and eventually how) the therapeutic approach should change in patients with IPF and lung cancer is controversial, and the guidelines make no specific recommendation in this regard [1]. Our approach is to make decisions on a case-by-case basis after careful evaluation of benefits and risks. Indeed, while IPF is not an absolute contraindication for lung cancer treatment, acute disease worsening has been reported following tumor resection, radiation therapy, radiofrequency ablation, and chemotherapy [56, 58]. Furthermore, chemotherapy-related adverse events may potentially affect the patient's ability to tolerate antifibrotic therapy.

### 3.5. How Do We Treat Elderly Patients?

While there is no general agreement on the age at which a person becomes “old,” in most developed countries persons achieving age 65 years or older are generally defined as elderly. IPF is strongly associated with advanced age and is highly prevalent in the elderly [3, 4]. The prognosis of IPF is also significantly worse in elderly patients than in younger patients [14]. Likely contributors to such adverse outcome include a more aggressive disease course, lead-time bias with or without delayed diagnosis, and comorbidities/complications that tend to be more prevalent and severe in this age group (e.g., coronary artery disease, sleep-disordered breathing, anxiety and depression, deconditioning, osteoporosis, and diabetes) [58, 59]. Moreover, interpretation of lung function testing (particularly 6MWT) in this age group may be challenging and should always be placed in the context of concomitant conditions. For instance, in elderly patients with walk-limiting musculoskeletal disease or significant frailty with sarcopenia, 6MWT may not be as useful as in younger patients [59–61].

Pirfenidone and nintedanib are generally safe and well tolerated, but significant side effects can also occur [18, 19]. Precautions should therefore be used, particularly in elderly patients taking other medications that may inhibit or induce the hepatic enzyme systems (e.g., CYP1A2, CYP3A4, and P-glycoprotein) that metabolize these two drugs. While in the INPULSIS trials there was no upper age limit for inclusion in the study [19], in both the CAPACITY and ASCEND trials patients older than 80 years of age were not enrolled [18, 27]. However, a post hoc analysis of pooled data from the CAPACITY and ASCEND trials showed that pirfenidone had similar efficacy and safety across different age subgroups (e.g., <65, 65 to 74, or ≥74 years) [62]. Management of elderly patients with IPF should be as personalized as possible in order to prevent functional decline and disease progression but also limit the risk of treatment-related adverse events [59].

## 4. Future Perspectives

Having two drugs available will lead many physicians to consider combination therapy, an approach that has been successfully applied to various respiratory diseases such asthma, chronic obstructive pulmonary disease, PAH, and lung cancer. Indeed, owing to the multifactorial and heterogeneous nature of IPF and the need to target simultaneously a multitude of overlapping profibrotic pathways, combination therapy might be a possibility. The add-on approach is more likely to be successful if drugs with established efficacy are used in combination, although a reasonable alternative could be the addition of a compound sufficiently promising to be evaluated clinically to a drug with established efficacy as background therapy (e.g., pirfenidone* or* nintedanib) [63]. While combining two drugs may produce a beneficial synergistic or additive effect, a weaker effect than expected (either because the mechanism of efficacy, whatever it might be, is targeted by both drugs and a “ceiling effect” is achieved, or because there is a blocking interaction) or an unpredictable interaction that drives disease progression or produces unacceptable side effects cannot be excluded. Therefore, combination therapy should be avoided until data are available demonstrating its efficacy and tolerability/safety in patients with IPF [50]. Recently, a randomized, double-blind, placebo-controlled phase II, dose escalation trial has been conducted to assess the safety, tolerability, and pharmacokinetics of nintedanib, alone and when added to ongoing pirfenidone therapy in Japanese patients with IPF [64]. Investigators observed a trend toward lower exposure of nintedanib when added to pirfenidone and suggested that further study is needed to evaluate the safety and tolerability profile of pirfenidone and nintedanib combination in patients with IPF [64].

The notion of “personalized” or “precision” medicine is based on the potential of customizing healthcare (e.g., determining disease susceptibility, delivering timely prevention, predicting outcome, and tailoring the right treatment for the right patient at the right time) by using molecular profiling technologies [65]. This approach has been successfully used to guide treatment decisions in patients with lung cancer (e.g., carriers of mutations within* EGFR*,* ALK*, and* K-RAS* genes have the best chance of responding to dedicated targeted therapies). In IPF, like in other complex multipathway diseases, developments in personalized care could potentially allow the identification of patient subgroups selectively (or more) responsive to treatment [66]. However, because of the multitude of signaling pathways involved simultaneously in the fibrotic process of IPF, targeted therapies are unlikely to be truly effective in isolation. Moreover, while personalized medicine relies on specificity of effect, both pirfenidone and nintedanib have multiple targets/effects [63]. Combination therapy and personalized medicine are, in principle, not mutually exclusive.

Another unsolved issue relates to the management of patients with* possible* or* probable* IPF. In fact, in clinical practice, a significant minority of patients (e.g., mainly those unable or unwilling to undergo surgical lung biopsy) cannot be diagnosed according to current guidelines [1]. Recent data suggest that a confident diagnosis can be reached in such patients providing clinical and radiological data are discussed and interpreted by an experienced multidisciplinary team [67–71]. Until this diagnostic approach is validated prospectively, we recommend patients with* possible* or* probable* IPF be referred to expert centers for further management.

## 5. Conclusions

IPF is a progressive and almost invariably deadly disease. Over the last decade, our understanding of the mechanisms involved in disease pathobiology has substantially improved. Similarly, disease definition and diagnostic criteria have been refined and this had allowed a number of high-quality clinical trials to be undertaken and completed. This massive effort of the medical and pharmaceutical community has led to the approval of two drugs for IPF, pirfenidone and nintedanib, which means that we may finally have choices for the pharmacological treatment of our patients with IPF. Yet, there is a long way still to go. Indeed, despite significant effect of antifibrotic therapy on functional decline, a cure for IPF has yet to be discovered. These limitations notwithstanding, there is genuine optimism that the concerted effort by the scientific, professional, and patient community as well as the pharmaceutical industry will lead soon to the development of a real cure for patients suffering from this devastating disease.

## Figures and Tables

**Table 1:** Key recommendations on pharmacological treatment of IPF according to current guideline.

	2015 guideline	2011 guideline
Therapeutic agent		
Pirfenidone	Conditional recommendation for use^*∗*^	Weak recommendation against use
Nintedanib	Conditional recommendation for use	Not addressed
Antiacid therapy	Conditional recommendation for use	Weak recommendation for use
Phosphodiesterase-5 inhibitor (sildenafil)	Conditional recommendation against use	Not addressed
Dual endothelin receptor antagonists (bosentan, macitentan)	Conditional recommendation against use	Strong recommendation against use
N-acetylcysteine (NAC)	Conditional recommendation against use	Weak recommendation against use
Azathioprine + corticosteroids + NAC	Strong recommendation against use	Weak recommendation against use
Warfarin	Strong recommendation against use	Weak recommendation against use
Imatinib	Strong recommendation against use	Not addressed
Selective endothelin receptor antagonist (ambrisentan)	Strong recommendation against use	Not addressed

^*∗*^ *Conditional* recommendations are synonymous with *weak* recommendations.

**Table 2:** Interpretation of *strong* and *conditional* recommendations for stakeholders (patients, clinicians, and health care policy makers)^*∗*^.

Implications for	Strong recommendation	Conditional recommendation
Patients	Most individuals in this situation would want the recommended course of action, and only a small proportion would not.	The majority of individuals in this situation would want the suggested course of action, but many would not.

Clinicians	Most individuals should receive the intervention. Adherence to this recommendation according to the guideline could be used as a quality criterion or performance indicator. Formal decision aids are not likely to be needed to help individuals make decisions consistent with their values and preferences.	Recognize that different choices will be appropriate for individual patients and that you must help each patient arrive at a management decision consistent with his or her values and preferences. Decision aids may be useful in helping individuals to make decisions consistent with their values and preferences.

Policy makers	The recommendation can be adopted as policy in most situations.	Policy making will require substantial debate and involvement of various stakeholders.

^*∗*^Reproduced from [23].
